# Rapid Oxidation of the Hole Transport Layer in Perovskite Solar Cells by A Low-Temperature Plasma

**DOI:** 10.1038/s41598-018-36685-6

**Published:** 2019-01-24

**Authors:** Yumeng Wang, Hao Qu, Chunmei Zhang, Qiang Chen

**Affiliations:** Lab of Plasma Physics and Materials, Beijing Institute of Graphic Communication, Beijing, 102600 China

## Abstract

Herein we report a strategy of rapid oxidation of the hole transport layer (HTL) in perovskite solar cells by using oxygen/argon mixture plasma. This strategy offers a promising approach for simple manufacturing, mass production, and industrial applications. Compared to the conventional process of overnight oxidation, only ~10 s of oxygen/argon mixture plasma treatment is enough for the solar cell devices with FTO/ETL/perovskite/HTL/Au structure demonstrating a high power conversion efficiency. It is found that the high concentration of atomic oxygen generated in plasma oxidizing the HTL improves the conductivity and mobility, and therefore the process time is considerably shortened. This novel approach is compatible with continuous mass production, and it is suitable for the fabrication of large-area perovskite solar cells in the future.

## Introduction

Perovskite structure was discovered by Gustav Rose in 1839^1^, with the identification of the calcium titanate (CaTiO_3_) compound. Nowadays, organometal halide perovskites are widely studied for solar cells^2,3^. Miyasaka *et al*.^4^ first incorporated the perovskites into dye-sensitized solar cells in 2006, and they reported that using CH_3_NH_3_PbBr_3_ perovskite with mesoporous TiO_2_ as the absorber, the cells exhibited an efficiency of 2.2%^5^. Since then, perovskite solar cells gained tremendous interest in the research community, and the efficiency has been rapidly improved by using different structures and fabrication methods^6^.

**Figure 1:**
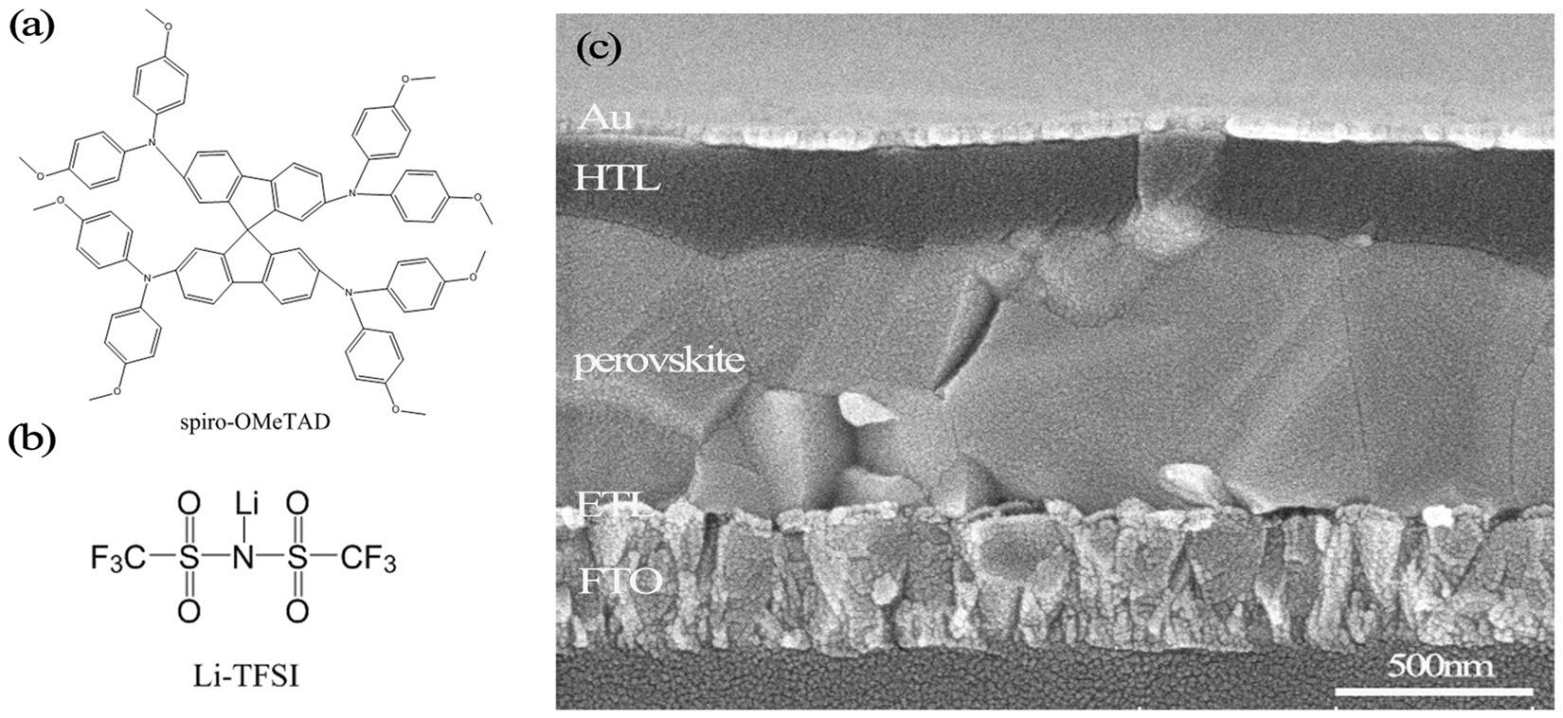
Molecular structures of (**a**) Spiro-OMeTAD and (**b**) Li-TFSI; (**c**) cross-sectional SEM image of a perovskite solar cell.

Normally, the perovskite solar cells are divided into three types based on their structures: mesoscopic perovskite solar cells, meso-superstructured perovskite solar cells, and planar heterojunction perovskite solar cells^7^. The structural simplification of the perovskite solar cells (PSCs), by replacing the mesoporous electron selective layer (ESL) with a planar one, is advantageous for large-scale manufacturing^8,9^. In the planar heterojunction perovskite solar cells, the perovskite is sandwiched between an electron selective layer (ESL, e.g., TiO_2_) and a hole transporting layer (HTL, e. g., doped 2,2′,7,7′-tetrakis(N,N′-di-p-methoxyphenylamine)-9,9′-spirobifluorene(Spiro-OMeTAD) or poly- teriary arylamine (PTAA)^10^. The molecular structure of Spiro-OMeTAD is shown in Fig. 1(a)^11^. This compound is amorphous, nonvolatile, and nonreactive with the perovskites, and it has a high melting point and a perfect band-gap matching to the perovskites, which makes Spiro-OMeTAD as one of the most popular HTL materials for PSCs^12,13^. However, the conductivity of Spiro-OMeTAD is not sufficiently high^14,15^. To this end, additives are doped into Spiro-OMeTAD. For instance, bis(trifluoromethane) -sulfonimide lithium salt (Li-TFSI, Fig. 1(b)) is often used as a *p*-type dopant to enhance the conductivity and hole mobility of Spiro-OMeTAD^16,17^. The lithium ions can facilitate the oxidation of Spiro- OMeTAD, and the large-size anions (TFS^−^) can stabilize the oxidized Spiro-OMeTAD as the counter-ions^18,19^. Unfortunately, the conventional oxidation process extends overnight, which is too lengthy for mass production and therefore needs considerable improvement.

Thus far, many methods have been studied to shorten the oxidation time of Spiro-OMeTAD, such as exposure in oxygen-containing environment^20^, the addition of other additives^21,22^, using a dicationic salt in Spiro-OMeTAD^23^, and O_3_ exposure under a UV light^24^. It was also noticed that the oxidation degree of Spiro-OMeTAD relied on the light intensity^25^, permeated oxygen concentration^26^, and Li^+^ ion concentration^27^. On the other hand, oxygen plasma is known of capability to generate highly reactive radicals, electrons, and ions, and it may improve the oxidation speed of Spiro-OMeTAD in perovskite solar cells. Therefore, we herein explore the approach of using oxygen plasma for HTL oxidization.

## Results and Discussion

The plasma setup used in this work was a capacitively coupled plasma (CCP), in which the upper plate was grounded, and the bottom plate was connected to the radio frequency (RF) power electrode, which was also used as a substrate holder. During the oxygen plasma treatment, the working pressure was constant at 20 Pa, and the 13.56 MHz RF power supply was set at 10 W.

**Figure 2:**
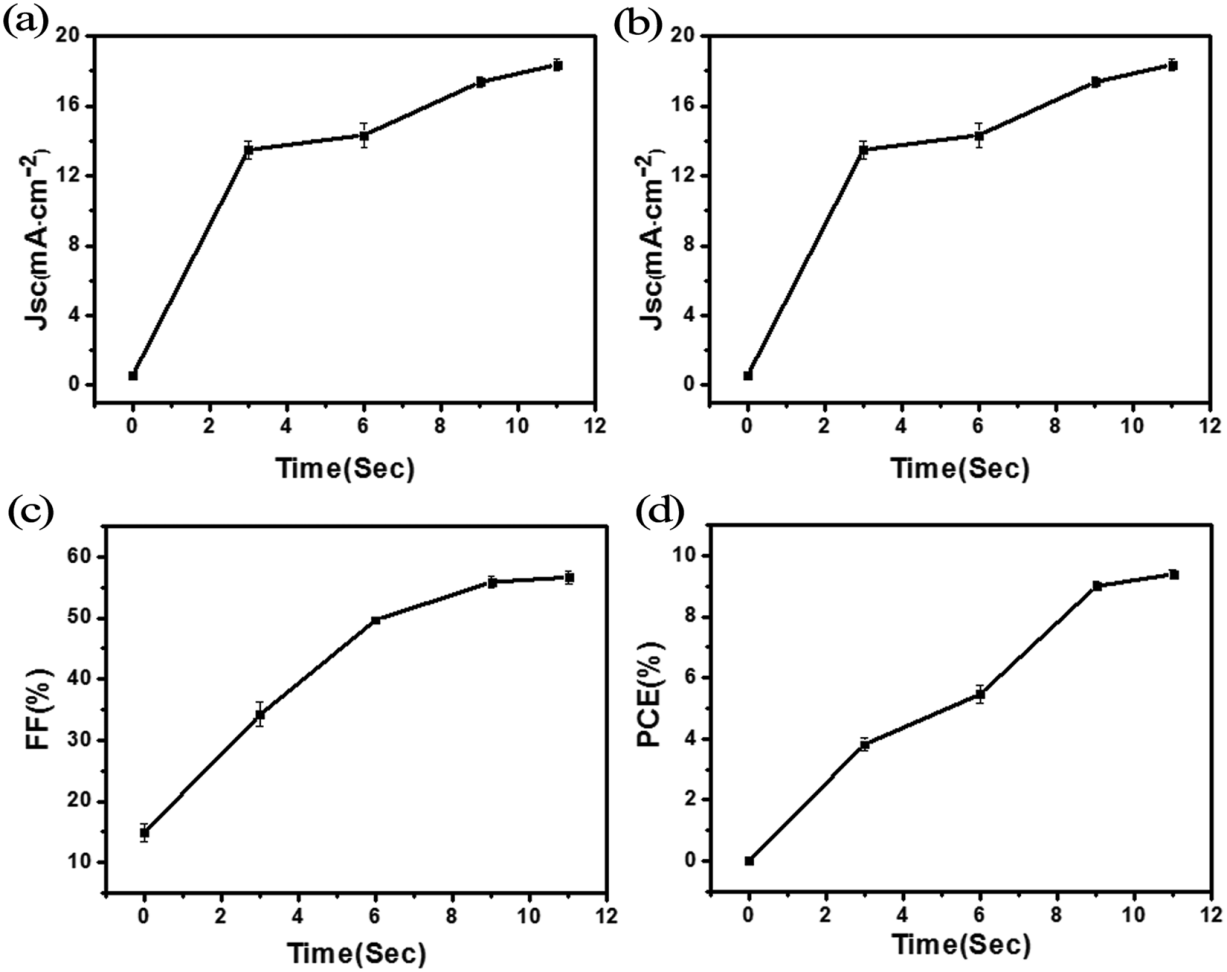
J_SC_ (**a**), V_OC_ (**b**), FF (**c**) and PCE (**d**) of the solar cells treated by oxygen plasma using various process times (from 0 to 11 s).

Figure 2 shows the performance of the PSCs after treated by the aforementioned oxidation process parameters, and Table 1 summarizes the device parameters averaged over five samples. It is founded that using the oxygen plasma treatment, and only 11 s was enough time for perovskite solar cells to exhibit good performance. Taking the 11 s treated samples as an example, compared to the untreated samples, the short-circuit current was increased from 0.56 to 18.37 mA/cm^2^, the open-circuit voltage was increased from 0.27 to 0.9 V, the fill factor was increased from 14.88% to 56.70%, and the power conversion efficiency (PCE) was increased from 0.01% to 9.40%. Compared to the control sample using conventional overnight oxidization process, of which the PCE was 12.73%, the 11 s plasma treated sample reached 74% of its efficiency.

**Table 1:** The averaged parameters of the perovskite solar cells with different plasma treatment times.

Time (s)	J_SC_ (mA/cm^2^)	V_OC_ (mV)	FF (%)	PCE (%)
0	0.56	0.27	14.88	0.01
3	13.48	0.78	34.17	3.82
6	14.34	0.80	49.70	5.46
9	17.37	0.91	55.91	8.99
11	18.37	0.9	56.70	9.40
12	3.40	0.07	6.39	0.01
overnight	18.89	0.97	69.60	12.75

We then analyzed the oxidized samples by UV-vis spectroscopy. Previous reports^27,28^ suggested that if the *p*-type dopant Spiro-OMeTAD was oxidized by molecular oxygen, and the optical absorption peak of the oxidized Spiro-OMeTAD was located at 400 nm, along with which an additional broad peak at around 500 nm appeared in the spectrum^29^. However, the 400 nm peak was usually indistinguishable because of a strong peak at 395 nm for the non-oxidized Spiro-OMeTAD, and therefore the broad peak at around 500 nm is generally used as the indicator of the oxidized Spiro-OMeTAD^19,30^.

**Figure 3:**
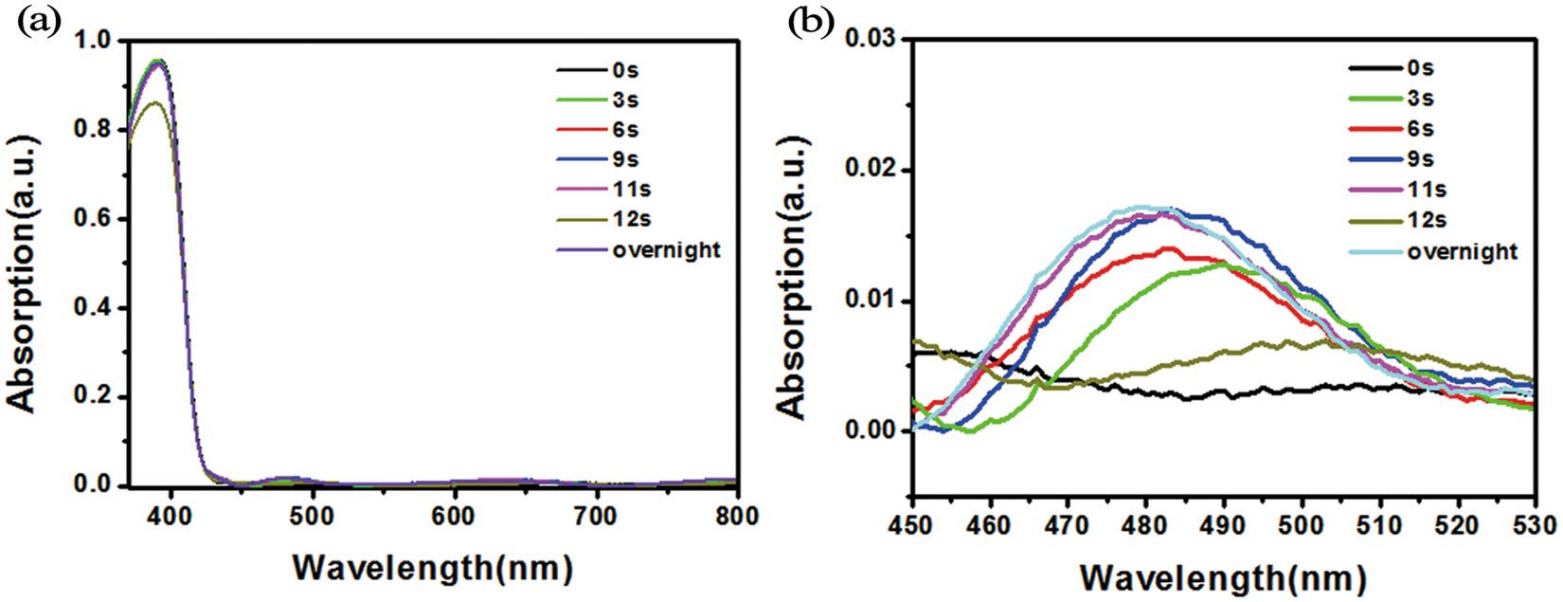
UV−vis spectra of the HTL using different plasma treatment times: (**a**) full range and (**b**) 460–630 nm range of the spectra.

Figure 3(a) shows the UV-vis spectrum of oxidized samples, along with which a spectrum for a pristine sample was also plotted for comparison. The appearance of the broad peak at around 500 nm indicated the indeed oxidization of HTL^31^. The peak in the range from 600 to 800 nm shifted with the increase of the process time, implies the higher oxidization degree of HTL with the treatment time^25^. Figure 3(b) shows the magnified peaks at around 500 nm. The 500 nm peak blue-shifted with the increase of the oxygen plasma exposure time was noticed, and this phenomenon was perhaps due to the slight variation of the HTL composition at different oxidation levels.

**Figure 4:**
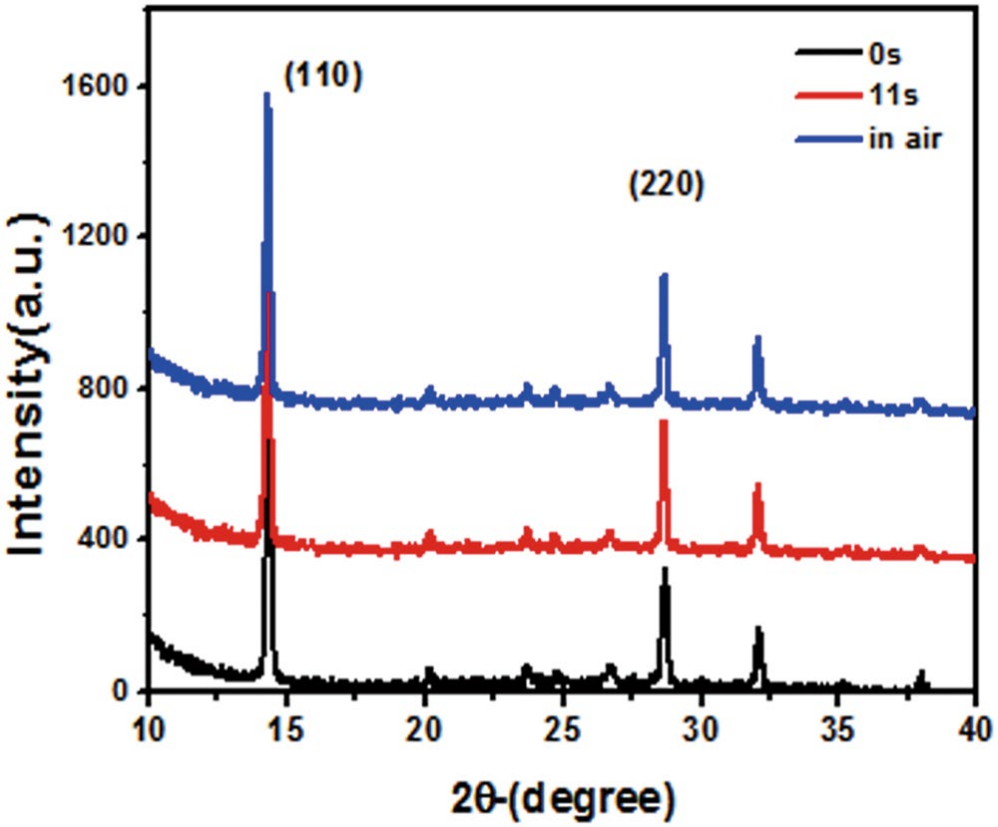
XRD patterns of the perovskite films after exposing in the inert gas, oxygen plasma, and dry air, respectively.

To further confirm that only the HTL was oxidized, not the perovskite layer, by the oxygen plasma, X-ray diffraction (XRD) measurement was carried out. We treated the samples in an inert gas atmosphere, dry air, and oxygen plasma, respectively, and then measured the XRD afterward. Figure 4 shows the XRD patterns of all samples, in which the peaks of (110) and (220) for perovskite film were visible. It infers that the perovskite layer was not affected by the plasma treatment. Similarly, another layers, such as inorganic FTO, TiO_2_, and Au, were also influenced little by plasma. Therefore, we think oxygen plasma did not influence the structure of the solar cells or might introduce non-detectable defects in the solar cells.

**Figure 5:**
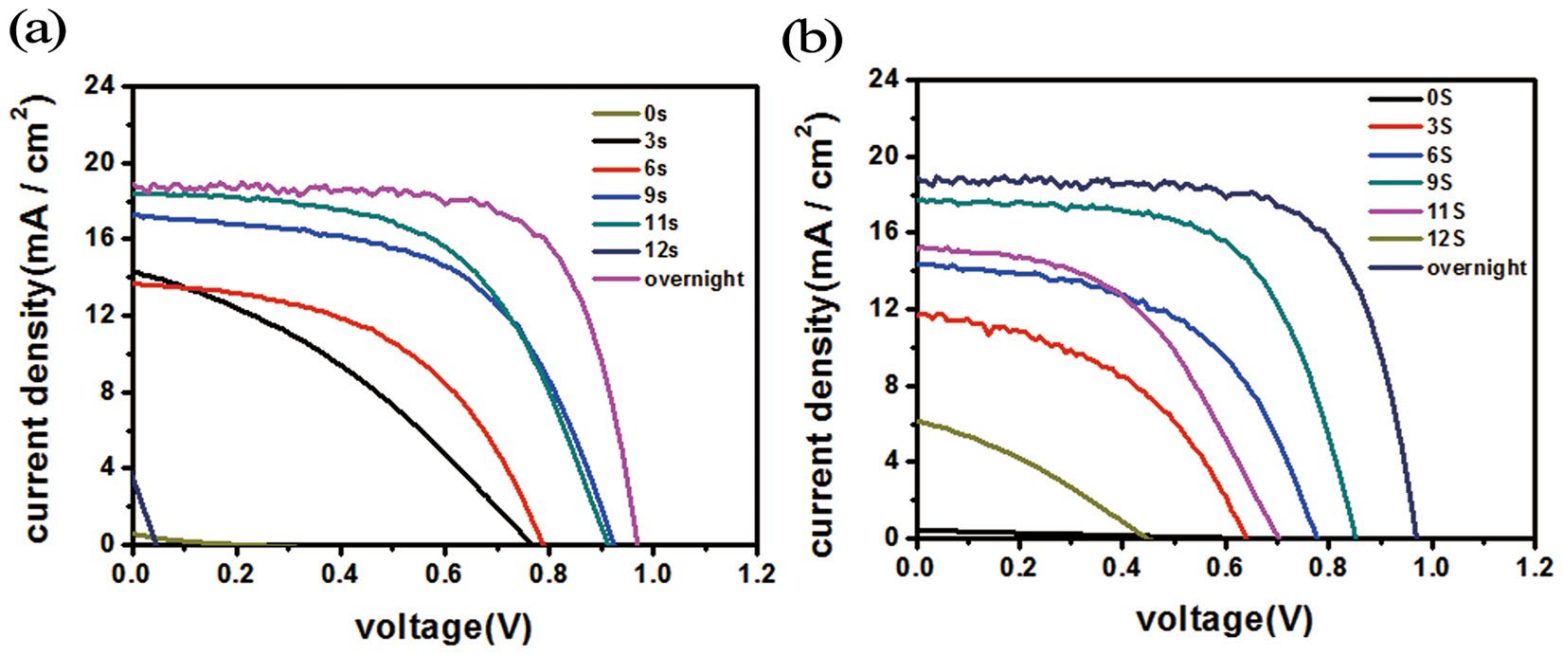
Comparison of the device performances for (**a**) the device oxidized samples and (**b**) the HTL oxidized samples.

We presume that the oxidization of the HTL in planar PSCs was likely caused by the diffusion of the oxidants generated in the oxygen plasma. Then we switched the sequence of the top-electrode (Au) deposition and the plasma treatment and compared this fabrication sequence effect. For the gold-deposition-first samples, the plasma was treated on the completed devices; whereas for the plasma-treatment-first samples, the plasma was directly treated on the HTL (Au was deposited after the plasma treatment). Figure 5 is the compared device performances. For the device oxidized samples, Fig. 5(a) and Table 2 show that the short-circuit current was increased from 0.58 to 18.41 mA/cm^2^, the open-circuit voltage was increased from 0.22 to 0.91 V, the fill factor was increased from 15.68% to 56.31%, and the power conversion efficiency was jumped from 0.02% to 9.43%; for the HTL oxidized samples, Fig. 5(b) and Table 3 show that the short-circuit current was increased from 0.43 to 17.73 mA/cm^2^, the open-circuit voltage was increased from 0.65 to 0.85 V, the fill factor was increased from 23.08% to 62.24%, and the power conversion efficiency was from 0.06% to 9.38%.

**Table 2:** The device parameters of perovskite solar cells versus the treatment time.

Time (s)	J_SC_ (mA/cm^2^)	V_OC_ (mV)	FF (%)	PCE (%)
0	0.58	0.22	15.68	0.02
3	14.32	0.77	34.42	3.80
6	13.65	0.79	49.51	5.34
9	17.23	0.92	56.56	8.97
11	18.41	0.91	56.31	9.43
12	3.39	0.05	6.36	0.01
overnight	18.90	0.97	69.41	12.73

**Table 3:** The device of perovskite solar cells without Au electrode versus the treatment time.

Time (s)	J_SC_ (mA/cm^2^)	V_OC_ (mV)	FF (%)	PCE (%)
0	0.43	0.65	23.08	0.06
3	11.69	0.64	45.82	3.43
6	11.54	0.81	59.58	5.57
9	17.73	0.85	62.24	9.38
11	15.13	0.70	48.67	5.15
12	6.11	0.45	31.85	0.88
overnight	18.90	0.97	69.41	12.73

Interestingly, the maximum PCE was achieved with 11 s plasma time for the device oxidized samples, but it was achieved with 9 s for the HTL oxidized samples. It implies that the oxidization of Li-TFSI in HTL was caused by the diffusion of the oxidants generated in the oxygen plasma. It is clear that at low pressure the diffusion speed of atomic oxygen in the devices was remarkably different in the devices with and without the top-electrode (Au). The atomic oxygen produced in plasma was definitely diffused quickly in HTL. As a result, the oxidation time was shorten.

**Figure 6:**
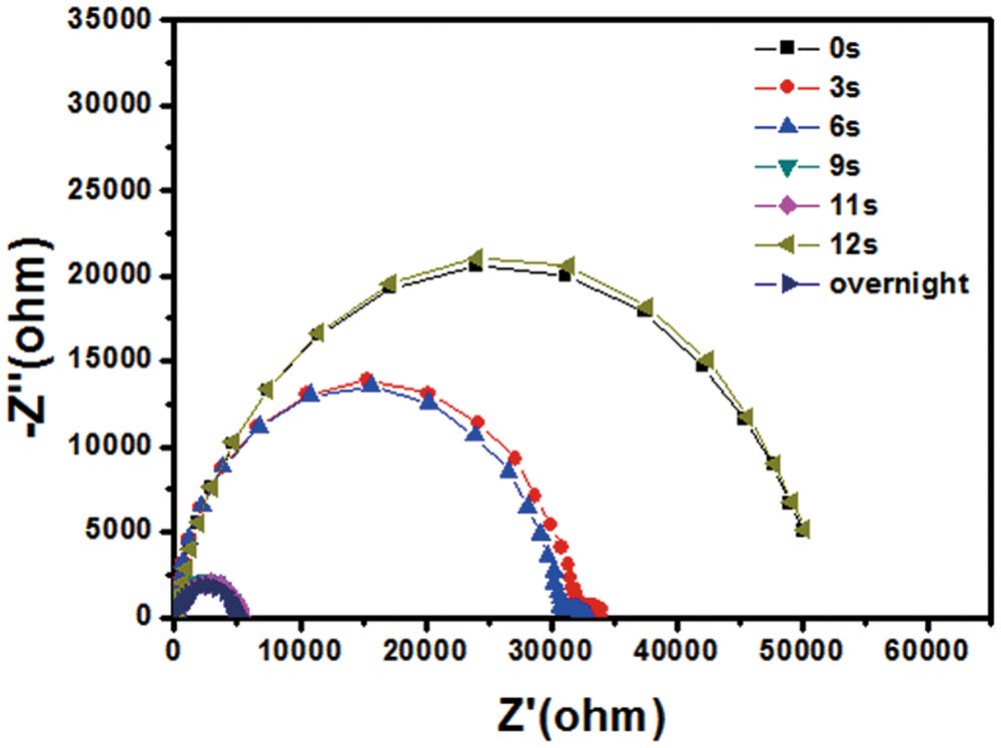
Nyquist plots of the HTL oxidized perovskite solar cells using different oxygen plasma treatment times.

Figure 6 shows the impedance spectroscopy (IS) results of the devices with the FTO/ETL/CH_3_NH_3_PbI_3_/HTL/Au structure. The most notable feature in Fig. 6 is the significant decrease of the impedance over the full range of frequency after the oxygen plasma treatment. The impedance of the perovskite solar cells could be satisfactorily modeled by an equivalent circuit consisting of a series resistor (R_S_) and a parallel combination of a resistor (R_rec_) and a capacitor^32,33^. In this circuit model, the fitted value of R_S_ is related to the internal resistance, and R_rec_ is associated with the interface charge transport process. After simulation, we found that R_S_ of the plasma-treated devices was smaller than that of the pristine device. Additionally, with the increase of the oxygen plasma treatment time, R_S_ gradually became smaller, which confirmed the oxidization of the doped Spiro-OMeTAD again because of the improved conductivity and mobility of HTL. However, when the oxygen plasma treatment time was over 11 s, the series resistance of the device was significantly increased, and the profile was similar to the untreated sample, which indicates that the device might have been damaged by over-oxidization^12^.

After analysis, we think that such significant change from 11 s to 12 s might be caused from the oxidation degree, because the oxidation activity of atomic oxygen is too strong, the structure of HTL was destroyed by excessive oxidation resulting in a marked increase in resistivity of the solar cell.

Both UV−vis spectrum of HTL and the device performance after 12 s O_2_ plasma treated devices support our conclusion. In UV−vis spectrum, it can be seen that the position of oxidation peak around 500 nm disappeared at 12 s treated device, which confirmed the ruination of HTL by the excessive oxidation.

Additionally, we found that, after several time repeated measurements of devices treated by 12 s oxygen plasma, the devices almost demonstrated no efficiency. This indicates that a long-term exposure to high concentration of the atomic oxygen, the oxidants will aggravate the deterioration of the device, resulting in rapid deterioration of device performance.

Furthermore, we consider that, at low pressure, the vacuum UV is very dense, which can quickly damage the organic matrix. The quantitative change to qualitative change by vacuum UV will appear even it extends a 1 s period.

**Figure 7:**
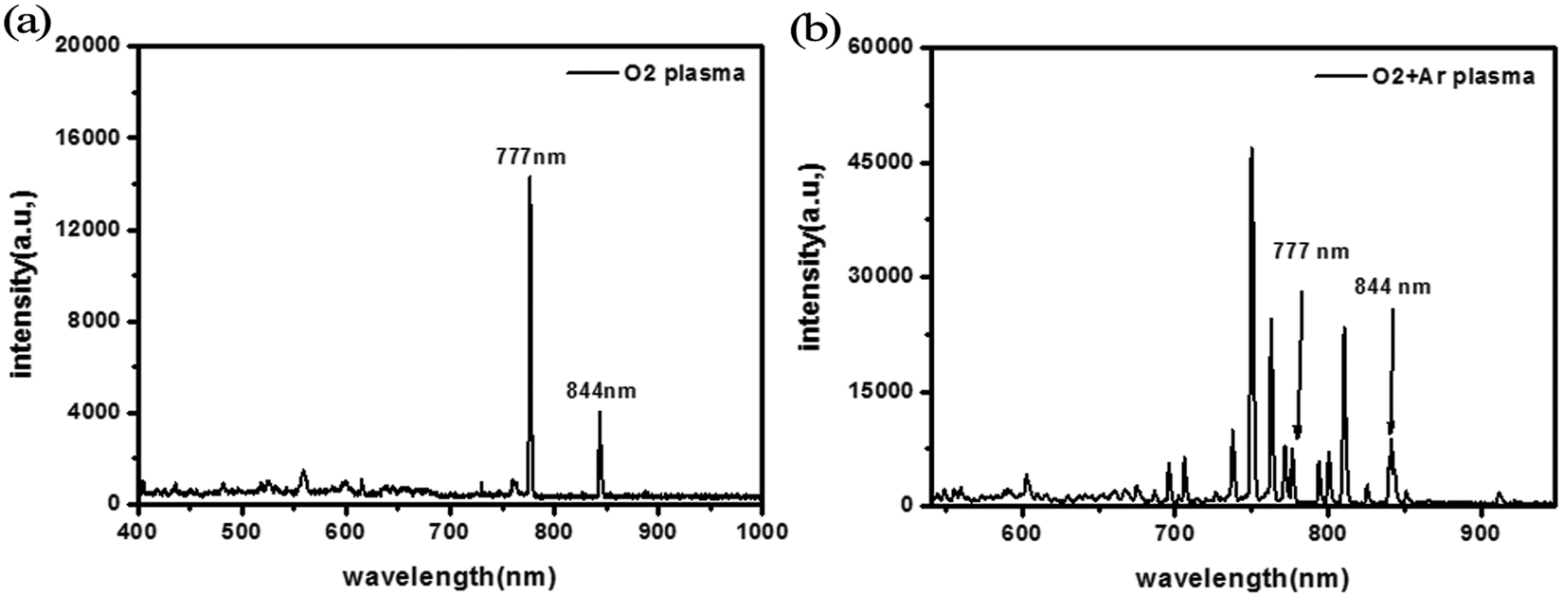
Survey OES of (**a**) the oxygen plasma and (**b**) oxygen/Ar mixtures plasma during the treatment of the solar cell devices.

To understand the mechanism of the oxygen plasma treatment, we measured the optical emission spectrum of the oxygen plasma. Figure 7(a) is the optical emission spectroscopy (OES) of capacitively coupled oxygen plasma. The wavelength of OES was scanned in the range from 200 to 850 nm, where the trigger time of the spectrometer was set to simultaneously measure spectra for each 200 ms. One can see that two atomic oxygen lines at 777 and 844 nm, corresponding to the transition O (3p5P → 3s5S) and O (3p3P → 3s3S), respectively, were quite strong in the spectrum, and no other strong peaks were observed in spectrum which indicates that the HTL was oxidized by the atomic oxygen generated in plasma, no molecular oxygen.

The main processes producing 777 and 844 nm lines are^34^: the dissociative excitation of molecule O_2_ and the direct electron impact excitation of molecule O_2_, respectively. The direct atomic excitation is more critical for 844 nm line, and the dissociative excitation is the dominant mechanism for line 777 nm^35^. To distinguish the role in the oxidation process, we flowed inert gas Ar into the oxygen plasma and compared the results with that of pure oxygen plasma.

Figure 7(b) and Table 4 show that by diluting oxygen with Ar, the intensity of the atomic oxygen at 777 nm exhibited noticeable change: the intensity of line 777 nm reduced by half from 14287.26 to 7451.78 whereas the intensity of line 844 nm showed only a slight decrease, i.e., plasma generated in Ar/oxygen mixture showed a smaller intensity of O (3p5P → 3s5S) for 777 nm.

**Table 4:** Intensity of the atomic oxygen lines at 777 and 844 nm in the optical emission spectrum of the plasma discharge.

Sample	777 nm	844 nm
O_2_ plasma	14287.26	3869.45
O_2_ + Ar plasma	7451.78	4789.7

**Figure 8:**
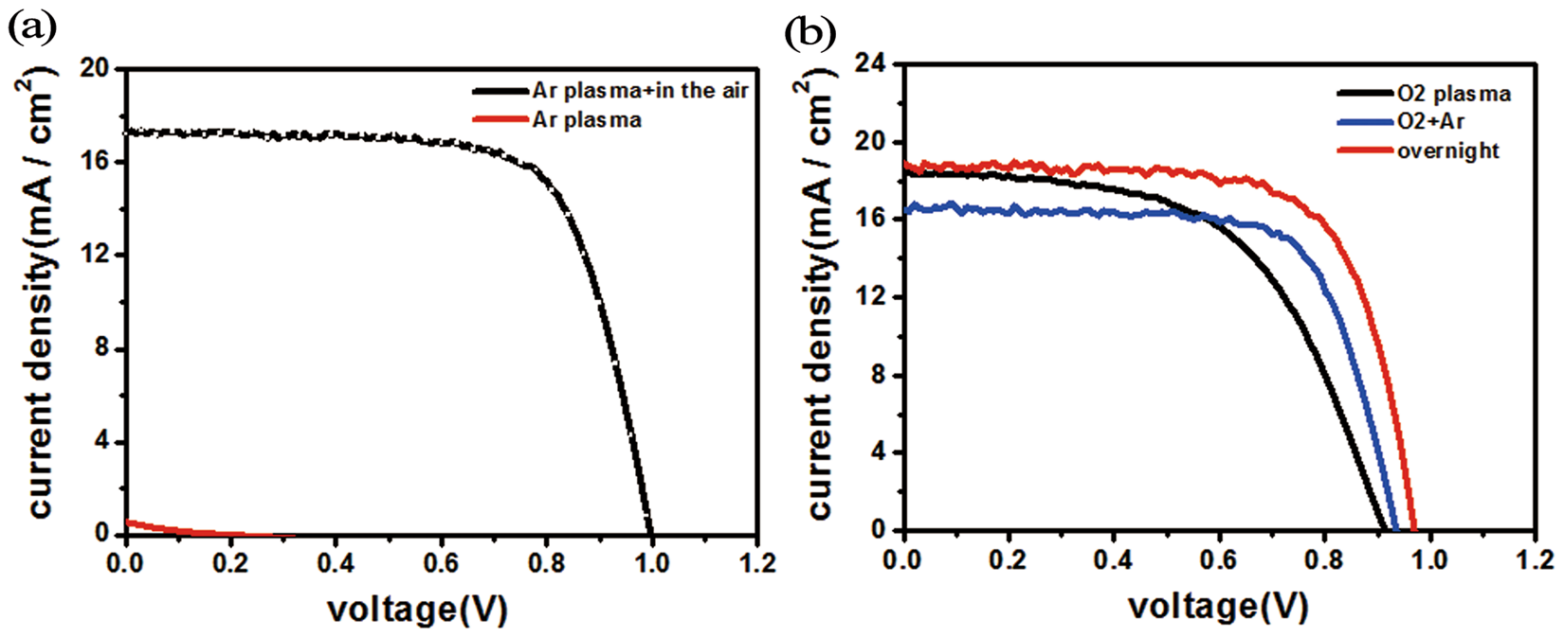
Comparison of the device performance of Ar plasma treated devices before (red) and after (**a**) overnight oxidation (black) and (**b**) mixed oxygen/Ar plasma.

After measuring the device performance, it was found that the Ar plasma treatment did not effect on the device efficiency. As shown in Fig. 8(a) and Table 5, the samples treated by Ar plasma did not have PCE at all. But if put the Ar plasma treated device in the air for overnight, the device efficiency was similar as the overnight oxidization sample.

**Table 5:** Comparison of the device performance of the perovskite solar cells treated by Ar plasma and then overnight oxidation.

Sample	J_SC_ (mA/cm^2^)	V_OC_ (mV)	FF (%)	PCE (%)
Ar plasma	0.58	0.22	15.68	0.02
Ar plasma + in air	17.21	1.00	71.03	12.22
O_2_ plasma	18.41	0.91	56.31	9.43
(O_2_ + Ar) plasma	16.53	0.93	71.49	10.99
overnight	18.90	0.97	69.41	12.73

In contrary, with the introduction of a small amount of Ar to dilute the oxygen plasma, the performance was much improved. Figure 8(b) and Table 5 show that for Ar diluted oxygen plasma treating sample the open-circuit voltage was increased from 0.91 to 0.93 V, the fill factor was increased from 56.31% to 71.49%, and the power conversion efficiency was increased from 9.43% to 10.99%. Therefore, we conclude that the atomic oxygen at 844 nm line, through dissociative excitations, played the crucial role in the HTL oxidization.

**Figure 9:**
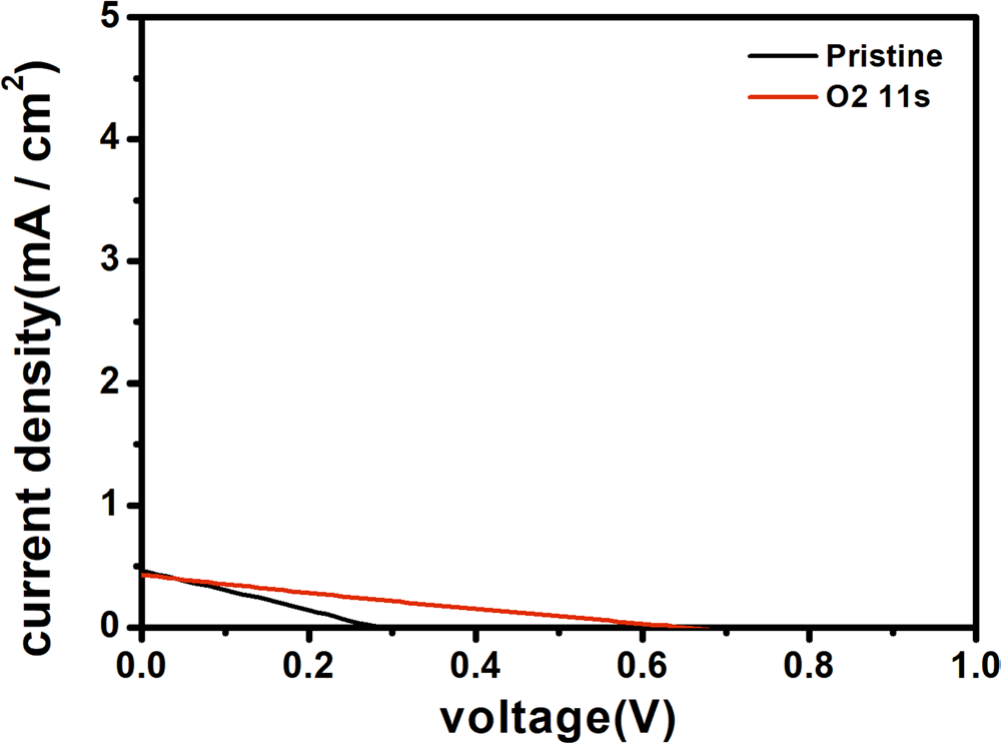
The device performance of oxygen molecule treated devices before (black) and after (red).

The remarkable increase of the conductivity has been exclusively ascribed to the electrostatic charge of the ionic species added into the organic matrix^18,36^. Abate *et al*.^19^ proposed a two-step oxidation mechanism by molecular oxygen. The reactions were written as:

1 $$Spiro-OMeTAD+{O}_{2}\leftrightarrow Spiro-OMeTA{D}^{\bullet +}\,{O}_{2}^{\bullet -}$$

2 $$Spiro-OMeTA{D}^{\bullet +}{O}_{2}^{\bullet -}+Li-TFSI\to Spiro-OMeTA{D}^{\bullet +}\,TFS{I}^{-}+L{i}_{x}{O}_{y}$$

First, the equilibrium between Spiro-OMeTAD with oxygen and oxidized Spiro-OMeTAD was established; second, the equilibrium moved forward by adding Li-TFSI, because the superoxide radical O^2−^ reacted with Li^+^ to form Li_x_O_y_ and finally generated Spiro-OMeTAD^·+^TFSI^−^.

In our work, we believe the atomic oxygen played the major role in the HTL oxidization, so it will be atomic oxygen joining the reaction, the oxidation mechanism was different from the Abate *et al*. equation. It is assumed that in the oxidation reactions, atomic oxygen replacing molecule oxygen to react with Li-TFSI, which serves either as a catalyst or a stabilizer of the oxidized Spiro-OMeTAD^16^. Therefore the reactions shall be written as:

3 $$Spiro-OMeTAD+2O\leftrightarrow Spiro-OMeTA{D}^{\bullet +}\,{(2O)}^{-}$$

4 $$Spiro-OMeTA{D}^{\bullet +}{(2O)}^{\bullet -}+Li-TFSI\to Spiro-OMeTA{D}^{\bullet +}\,TFS{I}^{-}+L{i}_{x}{O}_{y}$$

Additionally, in order to convince that the highly active atomic oxygen produced in the oxygen plasma quickly oxidize the HTL, we only input molecule oxygen in the chamber to treat the device. As expected no change of the device was observed after-treated in the molecule oxygen for 11 s as Fig. 9 shows.

## Conclusions

In this paper, we used CCP source to oxidize the HTL in perovskite solar cells with FTO/ETL/perovskite/HTL/Au structure. The solar cell PCE could be significantly improved after 11 s of oxygen plasma treatment, or 9 s oxygen/Ar mixture plasma treatment. Based on the characterizations by XRD, UV-vis spectroscopy, and impedance spectroscopy, it resulted that the oxygen plasma oxidized the HTL and improved its conductivity and mobility. Using OES diagnosis on the oxygen and oxygen/argon plasmas, we found that the Ar plasma did not affect the device performance, and the oxidization of the HTL was caused by atomic oxygen. The PCE reached 9.43% after ~11 s oxygen plasma treatment and increased to 10.99% after Ar/O_2_ plasma treatment. We believe that this strategy can greatly reduce the process time and make it suitable for large area devices and mass production.

## Materials and Methods

### Fabrication of perovskite solar cell

Fluorine-doped tin oxide (FTO) electrodes (sheet resistance 14Ω cm^−1^) were sequentially cleaned by ultrasonication in acetone (15 min), isopropanol (15 min), ethanol (15 min) and deionized water (15 min), respectively. Then the electrodes were dried by N_2_ stream and treated with ultraviolet light for 15 min. Compact TiO_2_ layer (TiO_2_) was deposited on FTO substrate by spin-coating an acidic titanium dioxide solution at 2000 rpm for 60 s. Then the substrate was annealed on a hotplate at 100 °C for 10 min, and sintered at 500 °C for 30 min.

The perovskite absorber layer was deposited on the compact TiO_2_ film by using a two-step spin coating method. The 460 mg of PbI_2_ in 1 mL N, N-Dimethyl formamide (DMF) mixed precursor solution was heated to 75 °C and magnetically stirred for 12 h simultaneously. The mixture solution was then spin-coated on the as-prepared FTO film at 1200 rpm for 30 s in a N_2_-filled glove box. Afterward, the as-prepared PbI_2_-based thin film was heated on a hot plate at 90 °C for 3 min in the glove box. The methyl ammonium iodide (MAI) solution including 40 mg CH_3_NH_3_I was dissolved in 1 mL isopropyl alcohol (IPA). The obtained MAI solution was spin-coated on the as-prepared thin film with the PbI_2_ complex at 6000 rpm for 60 s. HTL was then deposited via spin-coating a 0.8M solution of Spiro-MeOTAD in chlorobenzene, with additives of lithium bis (trifluoromethanesulfonyl) imide and 4-tert-butylpyridine. Spin-coating was carried out at 2000 rpm for 30 s. Lastly, an 80 nm thick gold was thermally deposited onto the film as a counter electrode.

## Characterizations

The current density-voltage (J-V) characteristics were gained by a Keithley 4200 source meter under the simulated light intensity of 100 mW/cm^2^ (ABET Technologies AM1.5). The obtained thickness was also verified by cross-sectional scanning electron microscopy (SEM) (Hitachi, Tokyo, Japan, SU8020). The light transmittance was obtained by ultraviolet visible spectrophotometer (UV-2501PC). Impedance spectroscopy measurements were carried out using a Bio-logic SAS equipped SP-150. Optical Emission Spectra (OES) measured by Ocean Optics(FLAME-S-XR1-ES). The film crystallinity was analyzed by x-ray diffraction (XRD) (Rigaku, Tokyo, Japan, D/max-2200 PC).
